# Predicting the eyebrow from the orbit using three-dimensional CT imaging in the application of forensic facial reconstruction and identification

**DOI:** 10.1038/s41598-023-30758-x

**Published:** 2023-03-10

**Authors:** Yi-Suk Kim, Won-Joon Lee, Ji-Su Yun, Dong-Ho Kim, Scott Lozanoff, U-Young Lee

**Affiliations:** 1 Catholic Institute for Applied Anatomy / Department of Anatomy, College of Medicine, The Catholic University of Korea, Seoul, 06591 Republic of Korea; 2 Division of Forensic Medicine, National Forensic Service Seoul Institute, Seoul, 08036 Republic of Korea; 3 Department of Forensic Medicine, National Forensic Service, Wonju, 26460 Republic of Korea; 4 Department of Anatomy, Biochemistry and Physiology, John A. Burns School of Medicine, University of Hawaiʻi at Mānoa, Honolulu, 96813 USA

**Keywords:** Anthropology, Biological anthropology, Anatomy, Skeleton

## Abstract

Eyebrows are the most important facial feature in facial recognition with its shape rated to be more helpful than color or density for facial reconstruction or approximation. However, little extant research has estimated the position and morphological territory of the eyebrow from the orbit. Three-dimensional craniofacial models, produced from CT scans of 180 Koreans autopsied at the National Forensic Service Seoul Institute, were used to conduct metric analyses of subjects (125 males and 55 females) between 19 and 49 (mean 35.1) years. We employed 18 craniofacial landmarks to examine the morphometry of the eyebrow and orbit with 35 pairs of distances per subject measured between landmark and reference planes. Additionally, we used linear regression analyses to predict eyebrow shape from the orbit for every possible combination of variables. The morphology of the orbit has more influence on the position of the superior margin of the eyebrow. In addition, the middle part of the eyebrow was more predictable. The highest point of the eyebrow in female was located more medially than the male. Based on our findings, the equations for estimating the position of the eyebrow from the shape of the orbit is useful information for face reconstruction or approximation.

## Introduction

Identifying the remains of a missing person, especially those whose faces cannot be recognized due to decomposition or skeletonization is often difficult for law enforcement and investigative agencies^1^. Moreover, this is especially true when evidence cannot be obtained from objective identifiable methods such as DNA, fingerprints, dental records, and non-dental radiographic comparison. Hence, facial reconstruction or facial approximation, a face recreation tool aimed to reproduce the face before death based on interpretation of the skull is employed, with the objective of recognition leading to an identification^2,3^. Furthermore, accuracy assessment also assists in the analysis of specific regions of the face, such as eyes, nose, mouth, and ears, which are critical to facial recognition by predicting the location, size, and morphology of facial features^4^.

Considerable data has been derived from craniofacial reconstruction studies in forensic identification. Fedosyutkin and Nainys, who summarized and described the relationship of skull morphology to facial features, showed general characteristics of how the skull morphology affects facial features^5^. The most frequently reported guidelines for facial feature properties is the study on the nose, such as nasal profile or projection^6–9^, followed by research on the eye, such as eyeball position or protrusion^10–12^, the mouth such as mouth width or lip morphology^13–15^, and ear shape estimation^16–18^. Conversely, Farkas et al. emphasized that facial morphology databases on various ethnic groups are still required^19^.

Among these features, eyebrows are the most important facial feature in recognizing emotions under the influence of cognitive load^20,21^. Specifically, eyebrow shape is more helpful than color or density in facial recognition^22^. However, to our knowledge, no research has been conducted that estimates the position and morphological territory of the eyebrow from the orbit using 3D craniofacial reconstruction methods. There have been studies of eyebrows using 2D methods and their importance has been described in face restoration with suggestions that additional studies be conducted to the point of the most superior part of the eyebrow^23^. This study establishes the parameters that may help estimate the position and shape of the eyebrow from the orbit using 3D computed tomography (CT) imaging methods. The findings of this study can be applied in the field of forensic facial reconstruction and are expected to increase the possibility of recognition and identification of persons.

## Results

In this study, 180 subjects were analyzed for each of the 35 measurements on both eyebrows and orbits. Descriptive statistics results are shown in Supplementary Table S2. In the intra- and inter-observer reliability analysis (effective N = 995), the alpha coefficients of each Cronbach were 0.999 and 0.998, showing very high reliability. In the analysis of sex difference by t-test, males showed significantly higher values than females in 23 of the 35 measurements. Descriptive statistical analyses represent the difference in the average value of each measurement section of the eyebrow and orbit between males and females obtained in the analysis. For the orbit, males had larger values for both width and height than females. In case of the eyebrow, lengths and heights of both sides were greater in males than females.

It has been reported that there is sexual dimorphism of eyebrows in primates^24^. Males had the larger mean value in the majority of measurements in this study. In males (Supplementary Table S3), the regression equation of measurement 14 with measurement 3 showed the highest power of explanation on both sides (R^2^ left 42%, right 47%). On the other hand, the regression equation taking measurement L10 to predict measurement L19 showed the lowest power of explanation (R^2^ 13%). On the right side, the equation predicting measurement R26 by measurement R9 showed the weakest power of explanation (R^2^ 18%). In females (Supplementary Table S4), the equation which predicts measurement L18 with measurement L9 showed the strongest power of explanation on the left side (R^2^ 35%). The highest power of explanation on the right side was shown by the equation for R10 making prediction of measurement R20 (R^2^ 43%). The measurement showing the lowest power of explanation on both sides was measurement 19 predicted by measurement 10 (R^2^ left 4%, right 5%). The measurements of the highest power of explanation differ in each sex. Therefore, to reconstruct a face, applying different measurements according to a subject’s sex would lead to a better outcome.

Further, we observed that there is discrepancy between sides (Supplementary Tables S3, S4). Measurements exhibiting the largest difference of power of explanation by sides in males were equation of measurement 19 by measurement 10 and equation of measurement 19 by measurement 8. On the left side, measurement 10 made a prediction of measurement 19 with R^2^ 13%; however, it was 31% on the right side. For the equation of measurement 19 by measurement 8, the power of explanation of the left side was 16% and the R^2^ was 34% on the right side. The discrepancies between sides were also observed in females. Regression equation of measurement 18 taking measurement 10 as a factor showed a R^2^ value of 28% on the left and 39% on the right. For the equation of measurement 21 estimated by measurement 9, the power of explanation was 21% on the left and 32% the right. R^2^ of the equation measurement 24 predicted by measurement 9 was 29% on the left side and 18% on the right side. Therefore, one might need to consider the side to apply the regression equation.

**Figure 1:**
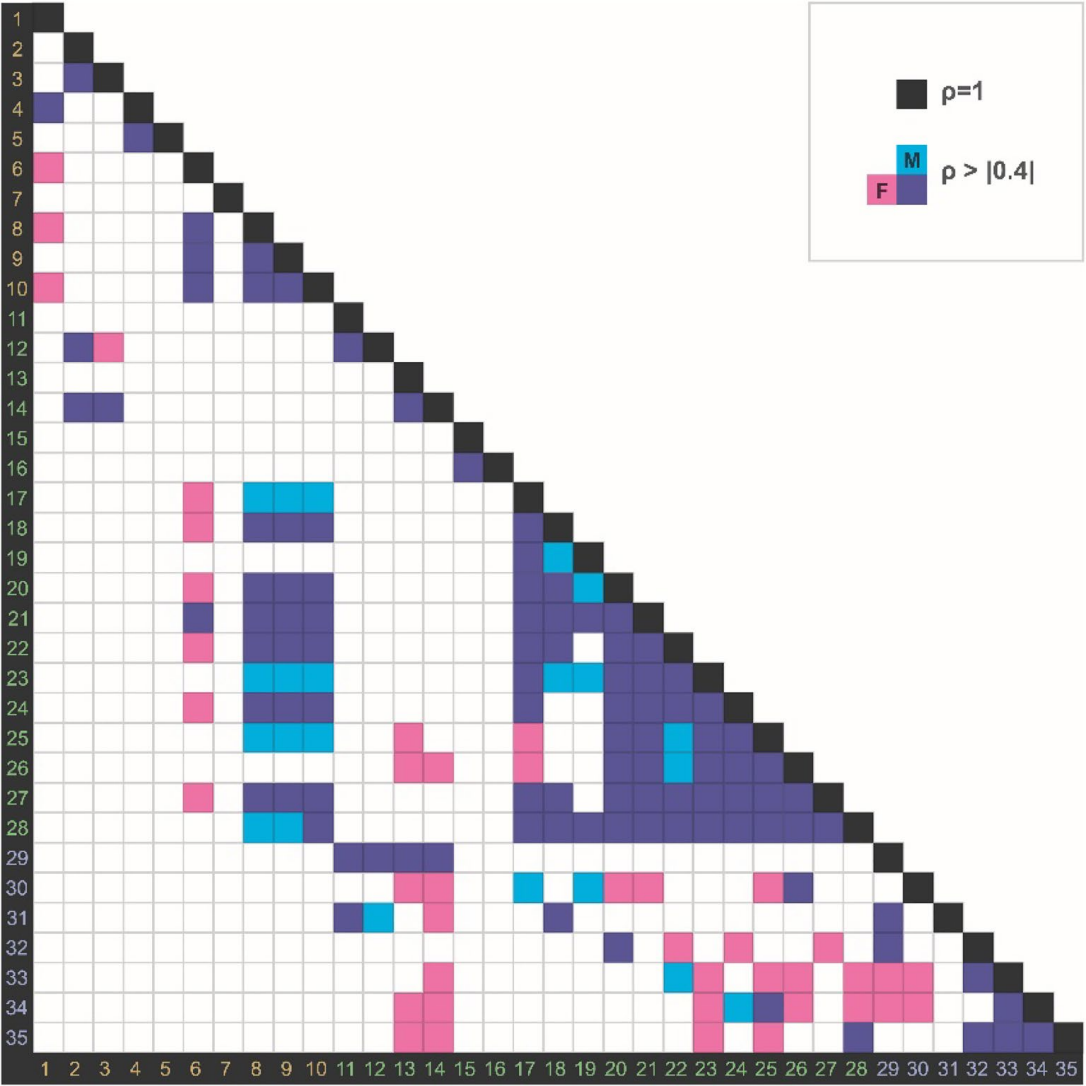
Summarized results of Pearson’s correlation coefficient in male (cyan) and female (magenta) groups. The cells represent correlations between measurement sections. Colored cells indicate correlation showing *ρ* >|0.4| on both left and right sides (*p* < 0.05).

The correlation coefficient increased as the height of the orbit and eyebrow was closer to the center of the orbit (Fig. 1). The height of the orbit (measurement code 8, 9, 10) showed a higher correlation with the height of the upper border (measurement code 17, 18, 20, 22, 24, 27) than the height of the lower border of the eyebrow (measurement code 19, 21, 23, 25, 26, 28).

All regression equations were developed from the measurements (Supplementary Tables S3, S4). Bivariate correlation analysis of pairs of bony and facial soft tissue sections in male and female groups revealed 14 pairs for males and females with Pearson’s correlation coefficients >|0.4|. Using the regression equations, the most effective prediction of eyebrow morphology was identified in Tables 1 and 2. In both sex, except for the medial and lateral heights of the eyebrow from orbitale (No. 17 and 26), height distances related to the inferior margin of the eyebrow (No. 19 and 25) in females; the coefficients for the regression equations were relatively high.

**Table 1:** Regression equations developed from the measurements in males.

Dependent variable	Independent variable	Regression equation	R^2^ (%)	Dependent variable	Independent variable	Regression equation	R^2^ (%)
L12	L2	L12 = 1.05 × L2 − 0.52	39	R12	R2	R12 = 1.05 × R2 − 0.85	43
L14	L3	L14 = 0.95 × L3 + 12.12	42	R14	R3	R14 = 1.06 × R3 + 9.27	47
L17	L8	L17 = 0.78 × L8 + 10.02	19	R17	R8	R17 = 1.02 × R8 + 0.03	28
L26	L8	L26 = 0.74 × L8 + 7.70	14	R26	R8	R26 = 0.84 × R8 + 4.48	19
L27	L8	L27 = 0.97 × L8 + 15.91	39	R27	R8	R27 = 0.93 × R8 + 17.32	37
L28	L8	L28 = 0.88 × L8 + 2.63	30	R28	R8	R28 = 0.88 × R8 + 2.75	31
L18	L8	L18 = 1.07 × L8 + 4.00	27	R18	R8	R18 = 1.16 × R8 − 1.13	35
L19	L9	L19 = 0.70 × L9 + 8.55	16	R19	R9	R19 = 1.16 × R9 − 8.58	32
L20	L8	L20 = 1.01 × L8 + 11.71	38	R20	R8	R20 = 1.06 × R8 + 9.55	44
L21	L8	L21 = 0.92 × L8 + 1.66	41	R21	R8	R21 = 0.99 × R8 − 0.87	44
L22	L10	L22 = 1.10 × L10 + 12.95	37	R22	R10	R22 = 1.05 × R10 + 14.30	37
L23	L10	L23 = 0.94 × L10 + 2.95	33	R23	R10	R23 = 1.01 × R10 + 0.27	35
L24	L10	L24 = 1.04 × L10 + 12.20	34	R24	R10	R24 = 0.92 × R10 + 15.88	28
L25	L10	L25 = 0.77 × L10 + 6.19	19	R25	R10	R25 = 0.95 × R10 − 0.02	26

The coefficient of determination is R^2^. All regression equations are shown in Supplementary Table S3.

‘L’ stands for left, and ‘R’ stands for right in each measurement.

**Table 2:** Regression equations developed from the measurements in females.

Dependent Variable	Independent Variable	Regression equation	R^2^ (%)	Dependent Variable	Independent Variable	Regression equation	R^2^ (%)
L12	L2	L12 = 0.71 × L2 + 3.97	29	R12	R2	R12 = 0.90 × R2 + 1.27	38
L14	L3	L14 = 0.83 × L3 + 11.58	32	R14	R3	R14 = 1.00 × R3 + 7.83	29
L17	L6	L17 = 0.66 × L6 + 23.40	22	R17	R6	R17 = 0.58 × R6 + 23.88	17
L26	L10	L26 = 0.97 × L10 + 2.07	13	R26	R10	R26 = 0.67 × R10 + 11.30	9
L27	L10	L27 = 1.00 × L10 + 13.46	33	R27	R10	R27 = 1.01 × R10 + 12.69	36
L28	L10	L28 = 0.97 × L10 + 1.02	24	R28	R10	R28 = 0.81 × R10 + 6.27	17
L18	L8	L18 = 0.88 × L8 + 8.38	34	R18	R8	R18 = 1.00 × R8 + 2.70	42
L19	L9	L19 = 0.36 × L9 + 18.64	4	R19	R9	R19 = 0.40 × R9 + 17.09	8
L20	L10	L20 = 0.99 × L10 + 11.40	34	R20	R10	R20 = 1.12 × R10 + 6.40	43
L21	L10	L21 = 0.72 × L10 + 8.76	26	R21	R10	R21 = 0.71 × R10 + 8.97	34
L22	L10	L22 = 0.96 × L10 + 14.17	32	R22	R10	R22 = 1.07 × R10 + 10.19	39
L23	L10	L23 = 0.86 × L10 + 4.55	20	R23	R10	R23 = 0.70 × R10 + 10.26	15
L24	L10	L24 = 1.11 × L10 + 6.53	34	R24	R10	R24 = 0.89 × R10 + 13.34	28
L25	L10	L25 = 0.81 × L10 + 5.91	11	R25	R10	R25 = 0.57 × R10 + 13.26	7

The coefficient of determination is R^2^. All regression equations are shown in Supplementary Table S4.

‘L’ stands for left, and ‘R’ stands for right in each measurement.

## Discussion

The eyebrow is a significant feature for recognition of a face. It is distinctive to the adjacent structures because eyebrows are located on superciliary ridges that protrude from the frontal bone. Further, the eyebrow is covered by eyebrow hair, which gives shade and texture to the region. The eyebrow functions as a factor to express emotions and recognize a face^25^.

The shape of eyebrows affects accuracy of the facial reconstruction. The eyebrow, however, wholly consists of soft tissue and prone to change its shape with plucking, shaving, and makeup in the living. These characteristics make estimation of eyebrows difficult in post-mortem face estimation. In former studies, the relationship of the eye structure and the eyebrow was researched. The location of the highest point of the eyebrow was explained in relation of the iris border and structures surrounding the eye using photographs of subjects with open eyes^23^. In this study, we considered whether measurement of the orbital rim is possible to estimate the shape of the eyebrow.

The general size of the orbit was larger in males; this is expected since the body size of males tends to be larger than females. Shape analysis, such as geometric morphometric analysis (GMM) has been applied to assess sex or ethnicity in other studies^26–28^. Future research should investigate sexual dimorphism and age change in Koreans using GMM, which was not dealt in this study. However, the landmarks used in this study would easily apply in GMM adding sliding landmarks between the landmarks from this study.

Every measurement except measurement 7 (LO-O), 16 (EBS-LO), and 25 (EB3I-O) was larger in males. The average of measurement 1 (MO-LO), a standard of measurement, is shorter in female. Therefore, EBS, the highest point of the eyebrow, is located more medial in females. The eyebrow of primates was reported to be sexually dimorphic, but the eyebrow solely as an indicator of sex has rarely been reported in humans. Stephan has suggested the average position of “superciliare,” the highest point of the eyebrow, for eyebrow reconstruction; however, he also noted that the application can be limited^23^. In Stephan’s study the mean horizontal distance between the superciliare and most lateral point of the iris showed a standard deviation greater than the mean. In this study, the R^2^ values ranged within 44%, suggesting a valid guideline, which provides a narrow-ranged figure, which can be worthwhile in facial reconstruction cases. However, caution should be taken in applying this method to other ancestries since this study was performed on Koreans.

## Conclusion

This study provided data to estimate the position of the eyebrow using the basic width and height measurement values of the orbit rim. The findings reveal that the morphology of the orbit had more influence on the position of the superior margin than the inferior margin of the eyebrow. In addition, the middle part of the eyebrow was relatively more predictable when the regression equations were used; however, the medial and lateral ends of the eyebrow were not. Therefore, both ends of the eyebrow are barely affected by the morphology of the orbital margin. Most of the male eyebrows had larger values than the female. The highest point of the eyebrow in female was located more medially than in the male. However, this fact is not clear enough to show sexual dimorphism. Through this study, it is expected that the equations for estimating the position of eyebrow from the shape of the orbit would be used as useful information for face reconstruction or approximation.

## Methods

### Samples and measurements

All methods performed in this study complied with the Declaration of Helsinki and were approved by the Institutional Review Board (IRB) of the National Forensic Service (No. 906-170118-HR-004-01). This retrospective study was approved, and prior informed consent was waived by Ethical Committee for National Forensic Service. The study is in accordance with relevant guidelines and regulations. The subject of all figures included in this study were images of Dr. Lee (Won-Joon Lee), a co-author and one of study participants, and written informed consent was obtained from him. We used craniofacial samples from 180 Koreans autopsied between March 2017 and September 2018 at the National Forensic Service Seoul Institute (NFS Seoul Institute). We conducted metric analyses for 180 subjects (125 males and 55 females) between the ages of 19 and 49 (mean, 35.1) years to minimize the influence of changes in eyebrow morphology due to aging (Table 3). We divided the subjects into six groups according to sex and age. All subjects arrived at NFS Seoul Institute within 48 h of death. Subjects with marked changes in the morphology of the head or face due to illness or the cause of death were excluded, as were individuals with congenital malformations or prosthetics in their eyebrow and orbit areas.

**Table 3:** Age distribution of samples.

Sex	Age (years)			Total
	19–29	30–39	40–49	
Male	21 (11.7%)	35 (19.4%)	69 (38.3%)	125 (69.4%)
Female	14 (7.8%)	20 (11.1%)	21 (11.7%)	55 (30.6%)
Total	35 (19.4%)	55 (30.6%)	90 (50.0%)	180 (100%)

The subjects were scanned using a SOMATOM Definition AS + (Siemens Healthineers, Erlangen, Germany). Barium sulfate (BaSO4) solution, a contrast agent, was applied to the subject’s eyebrows before taking the CT scans to reveal the radioactive area on the CT images. During this process, subjects with severe hair removal traces were excluded from the study. 3D craniofacial data were created using Digital Imaging and Communications in Medicine (DICOM) data acquired from a 128-slice multidetector CT (MDCT) scanner (SOMATOM Definition AS + , Siemens, Germany) under the following properties; 120 kV, 175 mA, and slice-thickness 0.6 mm. 3D models built using soft and hard tissue images were imported into a biomedical image engineering program (Mimics, version 20.0, Materialize, Leuven, Belgium), to obtain measurements of distances for 18 anatomical landmarks of the eyebrows and orbits.

**Figure 2:**
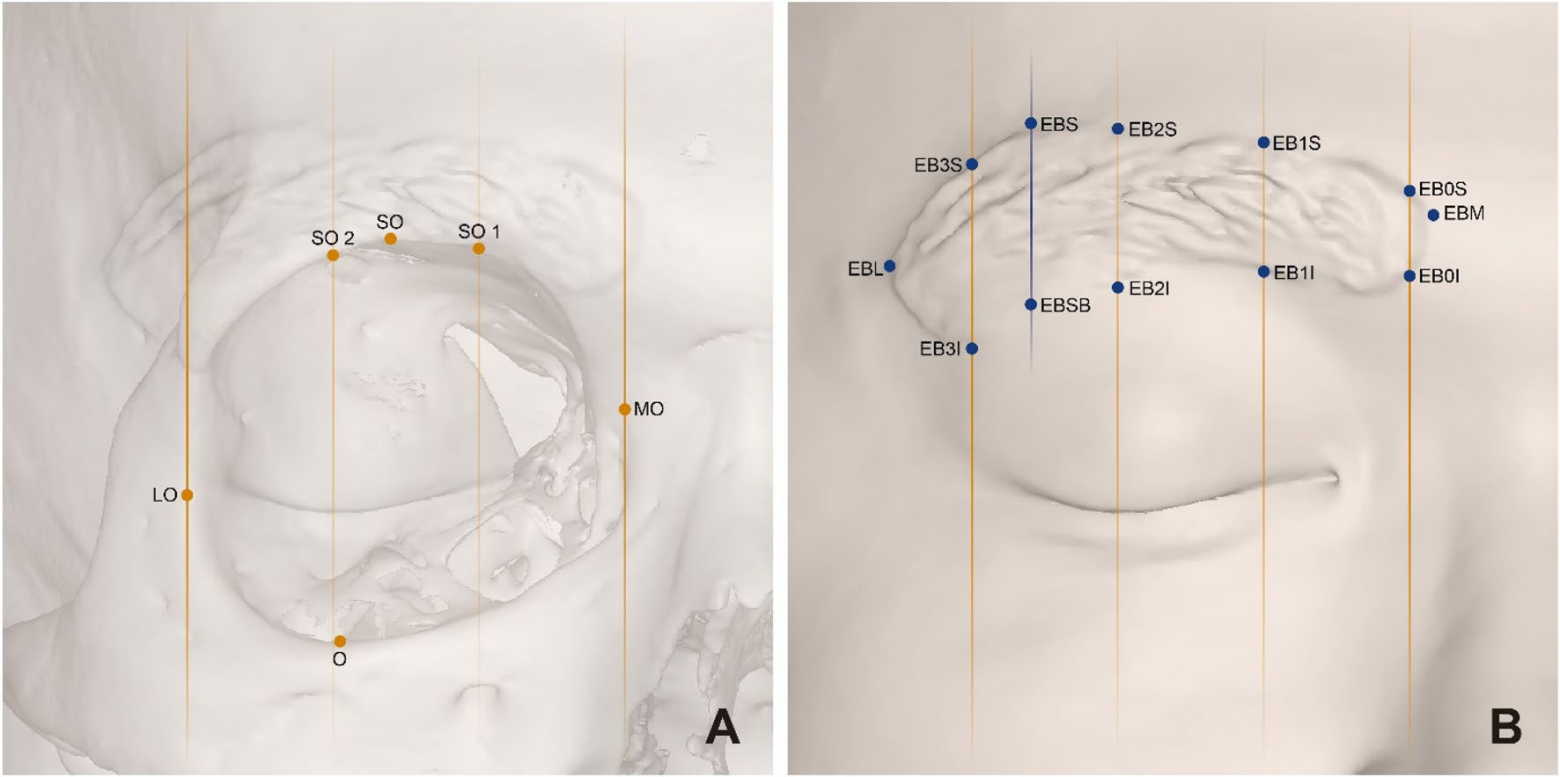
Anatomical landmarks of the orbit (**A**) and eyebrow (**B**). Detailed definitions of the landmarks are listed in Table 4.

**Figure 3:**
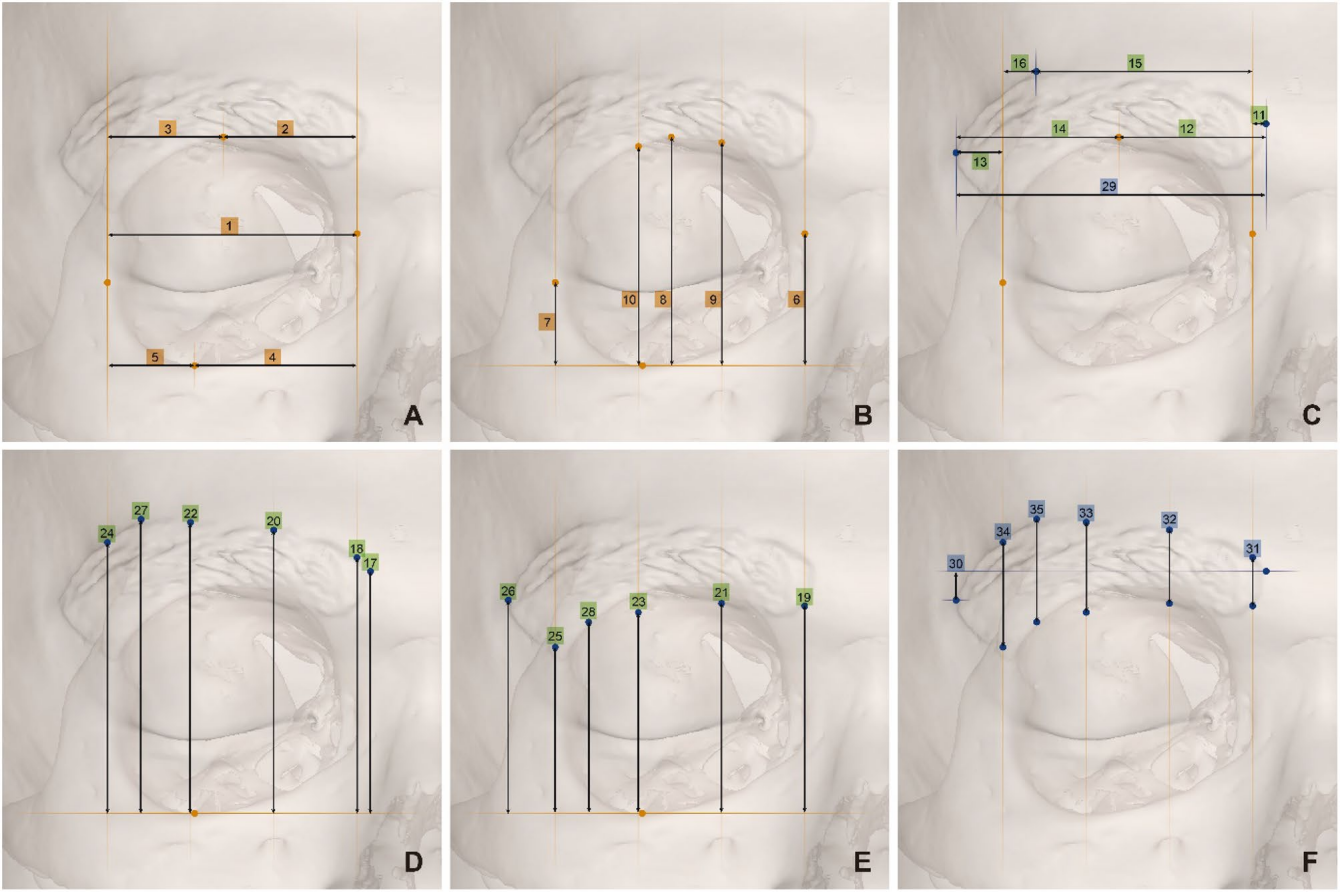
Thirty-five measurements between landmarks and reference planes (the numbers also indicate each measurement code; see Supplementary Table S1). Perpendicular distances between landmarks and reference planes. (**A**) Width measurements of the orbit; (**B**) height measurements of the orbit; (**C**) width measurements of the eyebrow; (**D**) height measurements of the upper border of the eyebrow; (**E**) height measurements of the lower border of the eyebrow; (**F**) height measurements of the eyebrows (orange colored dots are bony landmarks; blue colored dots are skin landmarks).

The Frankfort horizontal plane, a plane passing through orbitale and auriculare, as well as coronal and sagittal planes, perpendicular to each other were adopted as reference planes for cranio-cephalometric analysis. In total, 18 craniofacial landmarks [12 cephalometric (eyebrow) and 6 craniometric (orbit)] were used to examine the morphometry of the eyebrow and orbit (Table 4 and Fig. 2). The shortest distance from each reference plane (i.e., the perpendicular distance) was used as the position value of each landmark. We measured thirty-five pairs of distances between landmarks and reference planes per subject (Fig. 3; Supplementary Table S1).

**Table 4:** Variables and definitions of anatomical landmarks for the eyebrow and orbit.

Variables	Definitions
**Landmarks for alignment**	
Bregma (B)	Point where the sagittal and coronal sutures meet
Nasion (N)	Intersection of the nasofrontal sutures in the median plane
Prosthion (P)	Median point between the central incisors on the anterior most margin of the maxillary alveolar rim
Lambda (L)	Point at which the two legs of the lambdoid suture and sagittal suture meet (project from the main direction of the sutures in cases of obliteration or presence of wormian bones)
Auriculare (A)	On the zygomatic root, vertically above the center of the external auditory meatus
Auriculare midpoint (AM)	Median point of the line between left and right Au
Orbitale (Or)	Most inferior point on the inferior orbital rim. Usually falls along the lateral half of the orbital margin
**Landmarks for measurements**	
Orbitale (Or)	Most inferior point on the inferior orbital rim. Usually falls along the lateral half of the orbital margin
Medial orbit (MO)	Point on the anterior lacrimal crest at the same level as Ectoconchion
Lateral orbit (LO)	Most lateral point on the lateral orbital rim
Supraorbitale (SO)	Point on the anterior aspect of the superior orbital rim, at a line that vertically bisects the orbit
Supraorbitale-1 (SO1)	Point where the medial line among the two vertical lines trisecting the horizontal line where the MO and LO meet the superior orbital rim
Supraorbitale-2 (SO2)	Point where the lateral line among the two vertical lines trisecting the horizontal line where the MO and LO meet the superior orbital rim
Eyebrow-lateral (EBL)	Most lateral end point of the eyebrow
Eyebrow-medial (EBM)	Most medial end point of the eyebrow
Eyebrow-superior (EBS)	Most superior point on the superior margin of the unaltered eyebrow
Eyebrow-superior-basement (EBSB)	Meeting point of the inferior margin of the eyebrow and vertical passing line through the EBS
Eyebrow-0-superior (EB0S)	Meeting point on the vertical line of the MO and the superior margin of the eyebrow
Eyebrow-0-inferior (EB01)	Meeting point on the vertical line of the MO and the inferior margin of the eyebrow
Eyebrow-1-superior (EB1S)	One-third point between the MO and LO on the superior margin of the eyebrow from the medial side
Eyebrow-1-inferior (EB1I)	One-third point between the MO and LO on the superior margin of the eyebrow from the medial side
Eyebrow-2-superior (EB2S)	Two-thirds point between the MO and LO on the superior margin of the eyebrow from the medial side
Eyebrow-2-inferior (EB2I)	Two-thirds point between the MO and LO on the inferior margin of the eyebrow from the medial side
Eyebrow-3-superior (EB3S)	Meeting point on the vertical line of the LO and the superior margin of the eyebrow
Eyebrow-3-inferior (EB3I)	Meeting point on the vertical line of the LO and the inferior margin of the eyebrow

### Statistics

We conducted a statistical analysis using SPSS (version 21.0, SPSS, Chicago, IL, USA). Independent t-test and ANOVA were conducted after obtaining descriptive statistics for the samples, to verify significant differences between sex and age groups, respectively. We applied Levene’s test for homogeneity of variance under the assumption of equal variances (*p* > 0.05) as independent t-tests; otherwise, Mann–Whitney U-tests were used to determine sex differences. We also conducted intra-class correlation coefficient analysis to verify reproducibility of measurements by assessing intra- and inter-observer errors. Finally, we performed linear regression analyses using the SPSS to predict eyebrow shape from the orbit for every possible combination of variables using the command syntax. All statistical results were considered significant if p values were less than 0.05.

## Supplementary Information

Supplementary Information.

## Data Availability

The datasets generated during and/or analysed during the current study (expect CT images taken from the corpses) are available from the corresponding author on reasonable request.
