# Supplementary material for: Predicting the eyebrow from the orbit using three-dimensional CT imaging in the application of forensic facial reconstruction and identification
Source: Sci Rep. 2023 Mar 10;13:4014. doi: 10.1038/s41598-023-30758-x (PMC10006220; doi:10.1038/s41598-023-30758-x)
Supplement: Supplementary file 1 — Supplementary Information. [file 41598_2023_30758_MOESM1_ESM.docx]

**Supplementary Table 1**

Description of measurement sections in each measurement code

| Measurement code | Measurement section | | Measurement direction |
| --- | --- | --- | --- |
| 1 | MO-LO | Median Orbitale-Lateral Orbitale | Horizontal |
| 2 | MO-SO | Median Orbitale-Supraorbitale |  |
| 3 | LO-SO | Lateral Orbitale-Supraorbitale |  |
| 4 | MO-O | Median Orbitale-Orbitale |  |
| 5 | LO-O | Lateral Orbitale-Orbitale |  |
| 6 | MO-O | Median Orbitale-Orbitale | Vertical |
| 7 | LO-O | Lateral Orbitale-Orbitale |  |
| 8 | SO-O | Supraorbitale-Orbitale |  |
| 9 | SO1-O | Supraorbitale1-Orbitale |  |
| 10 | SO2-O | Supraorbitale2-Orbitale |  |
| 11 | EBM-MO | Eyebrow_medial-Median Orbitale | Horizontal |
| 12 | EBM-SO | Eyebrow_medial-Supraorbitale |  |
| 13 | EBL-LO | Eyebrow_lateral-Lateral Orbitale |  |
| 14 | EBL-SO | Eyebrow_lateral-Supraorbitale |  |
| 15 | EBS-MO | EBS-Median Orbitale |  |
| 16 | EBS-LO | EBS-Lateral Orbitale |  |
| 17 | EBM-O | Eyebrow_medial-Orbitale | Vertical |
| 18 | EB0S-O | EB0S-Orbitale |  |
| 19 | EBS0I-O | Eyebrow_3_inferior-Orbitale |  |
| 20 | EB1S-O | Eyebrow_1_superior-Orbitale |  |
| 21 | EB1I-O | Eyebrow_1_inferior-Orbitale |  |
| 22 | EB2S-O | Eyebrow_2_superior-Orbitale |  |
| 23 | EB2I-O | Eyebrow_2_inferior-Orbitale |  |
| 24 | EB3S-O | Eyebrow_3_superior-Orbitale |  |
| 25 | EB3I-O | Eyebrow_3_inferior-Orbitale |  |
| 26 | EBL-O | Eyebrow_lateral-Orbitale |  |
| 27 | EBS-O | EBS-Orbitale |  |
| 28 | EBSB-O | EBSB-Orbitale |  |
| 29 | EBM-EBL | Eyebrow_medial-Eyebrow_lateral | Horizontal |
| 30 | EBM-EBL | Eyebrow_medial-Eyebrow_lateral | Vertical |
| 31 | EB0S-EB0I | Eyebrow_0_superior-Eyebrow_0_inferior |  |
| 32 | EB1S-EB1I | Eyebrow_1_superior-Eyebrow_1_inferior |  |
| 33 | EB2S-EB2I | Eyebrow_2_superior-Eyebrow_2_inferior |  |
| 34 | EB3S-EB3I | Eyebrow_3_superior-Eyebrow_3_inferior |  |
| 35 | EBS-EBSB | Eyebrow_superior-Eyebrow_superior_basement |  |

**Supplementary Table 2**

Descriptive statistics

|  |  |  |  | Total (n=360) | | | | |  | Male (n=250) | | |  | Female (n=110) | | |  | Intra- observer | Inter- observer |
| --- | --- | --- | --- | --- | --- | --- | --- | --- | --- | --- | --- | --- | --- | --- | --- | --- | --- | --- | --- |
|  |  |  |  |  | Mean | SD | Min | Max |  |  | Mean | SD |  |  | Mean | SD |  | ICC | ICC |
|  | | | | |  |  |  |  |  |  |  |  |  |  |  |  |  |  |  |
| Hard tissue to hard tissue | distance (㎜) | 1 | MO-LO** |  | 40.31 | 2.03 | 35.44 | 45.63 |  |  | 40.92 | 1.92 |  |  | 38.95 | 1.54 |  | 0.99 | 0.99 |
|  |  | 2 | MO-SO |  | 17.07 | 2.56 | 9.62 | 24.79 |  |  | 17.21 | 2.54 |  |  | 16.76 | 2.58 |  | 0.93 | 0.80 |
|  |  | 3 | LO-SO** |  | 23.24 | 2.66 | 15.71 | 31.35 |  |  | 23.70 | 2.57 |  |  | 22.19 | 2.57 |  | 0.93 | 0.82 |
|  |  | 4 | MO-O** |  | 25.18 | 2.74 | 15.90 | 31.65 |  |  | 25.72 | 2.35 |  |  | 23.94 | 3.13 |  | 0.91 | 0.94 |
|  |  | 5 | LO-O |  | 15.14 | 2.01 | 10.92 | 21.88 |  |  | 15.19 | 1.89 |  |  | 15.01 | 2.26 |  | 0.90 | 0.93 |
|  |  |  |  |  |  |  |  |  |  |  |  |  |  |  |  |  |  |  |  |
|  | height (㎜) | 6 | MO-O** |  | 20.67 | 2.37 | 14.20 | 29.48 |  |  | 21.12 | 2.23 |  |  | 19.63 | 2.38 |  | 0.89 | 0.91 |
|  |  | 7 | LO-O |  | 16.12 | 3.06 | 9.56 | 23.37 |  |  | 16.01 | 3.06 |  |  | 16.38 | 3.05 |  | 0.95 | 0.96 |
|  |  | 8 | SO-O** |  | 36.09 | 2.10 | 30.37 | 42.34 |  |  | 36.37 | 2.11 |  |  | 35.47 | 1.94 |  | 0.99 | 0.99 |
|  |  | 9 | SO1-O** |  | 35.67 | 2.13 | 29.34 | 42.22 |  |  | 35.96 | 2.16 |  |  | 35.01 | 1.90 |  | 0.99 | 0.98 |
|  |  | 10 | SO2-O* |  | 34.09 | 1.89 | 28.97 | 40.05 |  |  | 34.25 | 1.87 |  |  | 33.73 | 1.88 |  | 1.00 | 1.00 |
|  |  |  |  |  |  |  |  |  |  |  |  |  |  |  |  |  |  |  |  |
|  |  |  |  |  |  |  |  |  |  |  |  |  |  |  |  |  |  |  |  |
| Hard tissue to soft tissue | distance (㎜) | 11 | EBM-MO**† |  | -0.10 | 3.12 | -8.22 | 12.52 |  |  | 0.14 | 3.18 |  |  | -0.63 | 2.95 |  | 0.99 | 0.99 |
|  |  | 12 | EBM-SO* |  | 16.99 | 4.01 | 6.66 | 29.06 |  |  | 17.37 | 4.13 |  |  | 16.13 | 3.57 |  | 0.97 | 0.90 |
|  |  | 13 | EBL-LO**†† |  | 9.86 | 3.36 | -4.31 | 22.72 |  |  | 10.78 | 2.87 |  |  | 7.77 | 3.47 |  | 0.99 | 0.98 |
|  |  | 14 | EBL-SO** |  | 33.10 | 4.47 | 18.90 | 46.48 |  |  | 34.48 | 3.86 |  |  | 29.95 | 4.15 |  | 0.97 | 0.93 |
|  |  | 15 | EBS-MO* |  | 28.55 | 6.04 | 8.09 | 42.78 |  |  | 29.28 | 6.17 |  |  | 26.90 | 5.41 |  | 0.97 | 0.51 |
|  |  | 16 | EBS-LO |  | 11.78 | 5.91 | 0.37 | 34.29 |  |  | 11.65 | 6.13 |  |  | 12.07 | 5.41 |  | 0.98 | 0.58 |
|  |  |  |  |  |  |  |  |  |  |  |  |  |  |  |  |  |  |  |  |
|  | height (㎜) | 17 | EBM-O** |  | 37.19 | 3.93 | 27.29 | 49.14 |  |  | 37.80 | 4.00 |  |  | 35.80 | 3.39 |  | 0.99 | 0.99 |
|  |  | 18 | EB0S-O** | (n=180) | 41.12 | 4.16 | 29.58 | 53.67 |  | (n=133) | 41.87 | 4.25 |  | (n=47) | 39.02 | 3.10 |  | 0.99 | 0.98 |
|  |  | 19 | EB0I-O** | (n=180) | 32.67 | 3.85 | 22.65 | 42.02 |  | (n=133) | 33.29 | 3.94 |  | (n=47) | 30.92 | 2.97 |  | 0.99 | 0.96 |
|  |  | 20 | EB1S-O** |  | 47.06 | 3.75 | 37.19 | 56.44 |  |  | 48.17 | 3.42 |  |  | 44.53 | 3.21 |  | 0.99 | 1.00 |
|  |  | 21 | EB1I-O** |  | 34.51 | 3.08 | 26.99 | 43.31 |  |  | 35.15 | 3.10 |  |  | 33.04 | 2.48 |  | 0.99 | 1.00 |
|  |  | 22 | EB2S-O** |  | 49.14 | 3.76 | 38.16 | 59.94 |  |  | 50.38 | 3.30 |  |  | 46.34 | 3.20 |  | 1.00 | 0.99 |
|  |  | 23 | EB2I-O** |  | 34.59 | 3.30 | 20.96 | 44.10 |  |  | 34.99 | 3.13 |  |  | 33.67 | 3.52 |  | 1.00 | 0.99 |
|  |  | 24 | EB3S-O** | (n=358) | 46.39 | 3.81 | 35.33 | 57.37 |  |  | 47.60 | 3.33 |  | (n=108) | 43.60 | 3.38 |  | 1.00 | 0.99 |
|  |  | 25 | EB3I-O | (n=358) | 32.58 | 3.69 | 19.81 | 43.34 |  |  | 32.48 | 3.40 |  | (n=108) | 32.81 | 4.29 |  | 0.98 | 0.99 |
|  |  | 26 | EBL-O |  | 34.55 | 4.25 | 18.85 | 46.96 |  |  | 34.66 | 4.09 |  |  | 34.29 | 4.60 |  | 0.97 | 0.99 |
|  |  | 27 | EBS-O** |  | 49.78 | 3.78 | 38.81 | 60.46 |  |  | 51.07 | 3.26 |  |  | 46.86 | 3.22 |  | 0.99 | 1.00 |
|  |  | 28 | EBSB-O |  | 34.42 | 3.49 | 22.42 | 43.08 |  |  | 34.75 | 3.36 |  |  | 33.66 | 3.68 |  | 0.98 | 0.99 |
|  |  |  |  |  |  |  |  |  |  |  |  |  |  |  |  |  |  |  |  |
|  |  |  |  |  |  |  |  |  |  |  |  |  |  |  |  |  |  |  |  |
| Soft tissue to soft tissue | distance (㎜) | 29 | EBM-EBL** |  | 50.09 | 5.34 | 34.42 | 68.03 |  |  | 51.85 | 4.61 |  |  | 46.09 | 4.70 |  | 1.00 | 0.99 |
|  |  |  |  |  |  |  |  |  |  |  |  |  |  |  |  |  |  |  |  |
|  | height (㎜) | 30 | EBM-EBL‡ |  | 2.65 | 4.72 | -13.57 | 18.17 |  |  | 3.14 | 4.87 |  |  | 1.51 | 4.16 |  | 0.98 | 0.98 |
|  |  | 31 | EB0 | (n=180) | 8.47 | 3.72 | 0.93 | 19.31 |  | (n=133) | 8.60 | 3.88 |  | (n=47) | 8.10 | 3.24 |  | 0.96 | 0.94 |
|  |  | 32 | EB1** |  | 12.55 | 2.21 | 6.55 | 19.68 |  |  | 13.02 | 2.09 |  |  | 11.48 | 2.12 |  | 0.97 | 0.92 |
|  |  | 33 | EB2** |  | 14.56 | 2.97 | 7.25 | 22.24 |  |  | 15.39 | 2.54 |  |  | 12.67 | 3.01 |  | 0.99 | 0.93 |
|  |  | 34 | EB3** | (n=358) | 13.82 | 3.86 | 1.96 | 23.65 |  |  | 15.13 | 3.03 |  | (n=108) | 10.79 | 3.90 |  | 0.97 | 0.97 |
|  |  | 35 | EBS-EBSB** |  | 15.37 | 3.16 | 7.46 | 26.57 |  |  | 16.32 | 2.72 |  |  | 13.20 | 3.02 |  | 0.96 | 0.99 |

*: p<0.05, **: p<0.001, significant differences between male and female; n=number of eyebrows

†: The negative values shown in the distance number 11 (EBM-MO) mean that the EBM is located internally (horizontally inside of the orbit) from the vertical line of passing the MO.

††: The negative value shown in the distance number 13 (EBL-LO) means that the EBL is located internally (horizontally inside of the orbit) from the vertical line of passing the SO.

‡: The negative value shown in the distance number 30 (EBM-EBL) means that the EBL is located inferiorly from the horizontal line of passing the EBM.

**Supplementary Table 3**

All regression equations developed from the measurements in the male group

| Regression equation | R² | Regression equation | R² |
| --- | --- | --- | --- |
| L12=1.05×L2-0.52 | 39% | R12=1.05×R2-0.85 | 43% |
| L14=0.95×L3+12.12 | 42% | R14=1.06×R3+9.27 | 47% |
| L17=0.78×L8+10.02 | 19% | R17=1.02×R8+0.03 | 28% |
| L17=0.77×L9+10.70 | 19% | R17=0.96×R9+2.47 | 27% |
| L17=0.82×L10+10.43 | 16% | R17=1.12×R10-1.12 | 27% |
| L26=0.74×L8+7.70 | 14% | R26=0.84×R8+4.48 | 19% |
| L26=0.73×L9+8.22 | 14% | R26=0.77×R9+7.25 | 18% |
| L26=0.82×L10+6.27 | 14% | R26=0.96×R10+2.12 | 20% |
| L27=0.97×L8+15.91 | 39% | R27=0.93×R8+17.32 | 37% |
| L27=0.94×L9+17.39 | 37% | R27=0.87×R9+19.85 | 35% |
| L27=1.09×L10+13.68 | 39% | R27=1.05×R10+14.92 | 38% |
| L28=0.88×L8+2.63 | 30% | R28=0.88×R8+2.75 | 31% |
| L28=0.86×L9+3.64 | 29% | R28=0.84×R9+4.73 | 30% |
| L28=0.95×L10+2.07 | 28% | R28=0.97×R10+1.43 | 30% |
| L18=1.07×L8+4.00 | 27% | R18=1.16×R8-1.13 | 35% |
| L18=1.05×L9+4.87 | 27% | R18=1.20×R9-1.80 | 36% |
| L18=1.20×L10+1.78 | 25% | R18=1.14×R10+2.15 | 29% |
| L19=0.71×L8+8.05 | 16% | R19=1.17×R8-9.56 | 34% |
| L19=0.70×L9+8.55 | 16% | R19=1.16×R9-8.58 | 32% |
| L19=0.74×L10+8.14 | 13% | R19=1.21×R10-8.58 | 31% |
| L20=1.01×L8+11.71 | 38% | R20=1.06×R8+9.55 | 44% |
| L20=0.98×L9+13.08 | 36% | R20=1.01×R9+11.72 | 44% |
| L20=1.15×L10+9.10 | 34% | R20=1.16×R10+8.12 | 43% |
| L21=0.92×L8+1.66 | 41% | R21=0.99×R8-0.87 | 44% |
| L21=0.91×L9+2.35 | 41% | R21=0.95×R9+1.03 | 43% |
| L21=1.02×L10+0.50 | 39% | R21=1.08×R10-1.88 | 41% |
| L22=0.96×L8+15.37 | 37% | R22=0.92×R8+16.87 | 36% |
| L22=0.94×L9+16.76 | 36% | R22=0.86×R9+19.44 | 34% |
| L22=1.10×L10+12.95 | 37% | R22=1.05×R10+14.30 | 37% |
| L23=0.84×L8+4.31 | 35% | R23=0.89×R8+2.62 | 34% |
| L23=0.83×L9+5.25 | 34% | R23=0.83×R9+5.08 | 32% |
| L23=0.94×L10+2.95 | 33% | R23=1.01×R10+0.27 | 35% |
| L24=0.91×L8+14.68 | 33% | R24=0.81×R8+17.95 | 27% |
| L24=0.88×L9+16.03 | 32% | R24=0.76×R9+20.22 | 25% |
| L24=1.04×L10+12.20 | 34% | R24=0.92×R10+15.88 | 28% |
| L25=0.65×L8-8.80 | 17% | R25=0.79×R8+3.80 | 23% |
| L25=0.64×L9-9.38 | 17% | R25=0.72×R9-6.49 | 20% |
| L25=0.77×L10+6.19 | 19% | R25=0.95×R10-0.02 | 26% |

‘L’ stands for left, and ‘R’ stands for right in each measurement.

**Supplementary Table 4**

All regression equations developed from the measurements in the female group

| Regression equation | R² | Regression equation | R² |
| --- | --- | --- | --- |
| L12=0.71×L2+3.97 | 29% | R12=0.90×R2+1.27 | 38% |
| L14=0.83×L3+11.58 | 32% | R14=1.00×R3+7.83 | 29% |
| L17=0.66×L6+23.40 | 22% | R17=0.58×R6+23.88 | 17% |
| L26=0.81×L8+6.12 | 10% | R26=0.65×R8+11.06 | 9% |
| L26=0.81×L9+6.12 | 10% | R26=0.65×R9+11.06 | 9% |
| L26=0.97×L10+2.07 | 13% | R26=0.67×R10+11.30 | 9% |
| L27=0.89×L8+15.40 | 28% | R27=0.87×R8+15.96 | 28% |
| L27=0.89×L9+15.83 | 28% | R27=0.84×R9+17.53 | 24% |
| L27=1.00×L10+13.46 | 33% | R27=1.01×R10+12.69 | 36% |
| L28=0.80×L8+5.51 | 18% | R28=0.69×R8+9.26 | 13% |
| L28=0.76×L9+7.19 | 18% | R28=0.70×R9+8.93 | 13% |
| L28=0.97×L10+1.02 | 24% | R28=0.81×R10+6.27 | 17% |
| L18=0.88×L8+8.38 | 34% | R18=1.00×R8+2.70 | 42% |
| L18=0.90×L9+7.96 | 35% | R18=1.04×R9+1.98 | 42% |
| L18=0.80×L10+12.80 | 28% | R18=0.97×R10+5.74 | 39% |
| L19=0.32×L8+19.78 | 4% | R19=0.32×R8+19.62 | 6% |
| L19=0.36×L9+18.64 | 4% | R19=0.40×R9+17.09 | 8% |
| L19=0.35×L10+19.51 | 4% | R19=0.30×R10+20.90 | 5% |
| L20=0.90×L8+12.80 | 31% | R20=0.99×R8+9.35 | 32% |
| L20=0.90×L9+13.12 | 31% | R20=0.98×R9+9.95 | 32% |
| L20=0.99×L10+11.40 | 34% | R20=1.12×R10+6.40 | 43% |
| L21=0.64×L8+10.55 | 22% | R21=0.67×R8+9.28 | 32% |
| L21=0.63×L9+11.16 | 21% | R21=0.70×R10+8.48 | 32% |
| L21=0.72×L10+8.76 | 26% | R21=0.71×R10+8.97 | 34% |
| L22=0.85×L8+16.46 | 27% | R22=0.93×R8+13.14 | 32% |
| L22=0.84×L9+16.98 | 26% | R22=0.90×R9+14.68 | 32% |
| L22=0.96×L10+14.17 | 32% | R22=1.07×R10+10.19 | 39% |
| L23=0.66×L8+10.19 | 12% | R23=0.56×R8+13.78 | 10% |
| L23=0.63×L9+11.72 | 12% | R23=0.56×R9+14.33 | 9% |
| L23=0.86×L10+4.55 | 20% | R23=0.70×R10+10.26 | 15% |
| L24=1.01×L8+8.12 | 30% | R24=0.78×R8+15.87 | 22% |
| L24=0.98×L9+9.62 | 29% | R24=0.74×R9+17.57 | 18% |
| L24=1.11×L10+6.53 | 34% | R24=0.89×R10+13.34 | 28% |
| L25=0.81×L10+5.91 | 11% | R25=0.57×R10+13.26 | 7% |

‘L’ stands for left, and ‘R’ stands for right in each measurement.
